# Non-IgE mediated food allergies in breastfed children: A clinical challenge 

**DOI:** 10.5414/ALX02364E

**Published:** 2022-10-05

**Authors:** Rosan Meyer, Imke Reese

**Affiliations:** 1 Department Medicine, Imperial College London, London,; 2 Department Nutrition and Dietetics, Winchester University, Winchester, Great Britain,; 3 Faculty of Medicine, KU Leuven, Belgium,; 4 Nutrition therapy, Munich, Germany

**Keywords:** non-IgE-mediated allergies, breast-fed infants, dietary elimination, diagnosis

## Abstract

The prevalence of non-immunoglobulin E (IgE) mediated food allergy is poorly established outside of cow’s milk allergy, with a challenge-proven adjusted incidence ranging between 0.13 and 0.72%. The presence and presentation of non-IgE mediated allergy in exclusively breastfed infants is highly debated. The dilemma this poses for healthcare professionals and parents, is on the one hand the unwarranted elimination and therefore health risk to the breastfeeding mother and on the other hand under-recognition of a food allergen being a culprit in the non-IgE mediated symptoms of breastfed infants. Current international guidelines recommend exclusive breastfeeding ideally until ~ 6 months of age and the German guidelines 4 – 6 months. It is also acknowledged that breastfeeding should be promoted also within the population of food-allergic infants. This review paper aims to assess non-IgE mediated food allergies in breastfed infants using an evidence-based approach and provides clinicians working with these patients with practical guidance.

## Introduction 

The prevalence of non-immunoglobulin E (IgE) mediated food allergy is poorly established outside of cow’s milk allergy (CMA). The EuroPrevall Study, based on over 12,000 infants from different European countries, found a challenge-proven adjusted incidence ranging between 0.13 and 0.72% [1]. In the United Kingdom, non-IgE mediated CMA was more prevalent than IgE mediated CMA, conversely, no non-IgE mediated CMA was reported in Iceland, Spain, Germany, Greece, and Lithuania [1]. The latter highlights an important problem with the current prevalence data on non-IgE mediated allergy, in that it is difficult to diagnose and often poorly recognized [2]. 

There is limited data of non-IgE mediated allergy involving other common allergens in children (i.e., soya, wheat, egg). Although this is reported in observational studies and healthcare professionals working in food allergy also have made this observation, the elimination of these food allergens is often based on experience rather than clinical research [3]. 

The presence of food allergy in exclusively breastfed infants remains a highly debated topic. The most commonly quoted study in breastfed infants is on CMA by Høst et al. [4] from 1988. This study found that 0.5% of the 2.2% children diagnosed with a challenge-proven IgE mediated CMA presented whilst being exclusively breastfed [4]. However, this is data on IgE mediated allergy and not non-IgE mediated allergy, but does imply plausibility of reactions occurring in breastfed infants. Current guidelines [5, 6, 7] recommend exclusive breastfeeding ideally until ~ 6 months of age [8], with German guidelines suggesting 4 – 6 months, and acknowledge that breastfeeding should be promoted also within the population of food-allergic infants. This paper aims to provide an evidence-based overview of the diagnosis and management of non-IgE mediated food allergy in breastfed infants. 

## Can food allergens through breastmilk lead to symptoms? 

At the heart of the debate of non-IgE mediated allergy in breastfed infants, is the question to whether maternally consumed food allergens can lead to symptoms. A recent scoping review found 27 studies assessing bovine milk protein in breastmilk that included data on the type of sampling method, the sampling time, the lactation stage, maternal allergy status, and, most importantly, the impact on the infant [9]. This publication found the presence of β-lactoglobulin, a bovine milk protein, in human breastmilk at similar levels previously published by Høst and Halken (range 0.9 – 150 µg/L) [9, 10]. Interestingly, they found that in some breastmilk samples, β-lactoglobulin was detected up to 7 – 10 days after stopping the consumption of cow’s milk [9]. This review found a significant correlation between high levels of β-lactogobulin in breast milk to clinical manifestations such as diarrhea, vomiting, colic, or eczema, which are all typical non-IgE mediated symptoms [11, 12]. Additionally, one study found that most of the infants with CMA reacted to cow’s milk challenge through human milk with the appearance of eczema in addition to, in some patients, diarrhea, abdominal pain, and regurgitation, at β-lactoglobulin level concentrations between 0.01 and 11.54 ng/mL [13]. 

The correlation of clinical manifestations and β-lactoglobulin does however not prove causality. This was argued by Munblit et al. [14] in 2020, who, based on a theoretical calculation, suggested that only 1 in 600 breastmilk samples, would contain sufficient β-lactoglobulin to elicit a reaction in 1% of the most sensitive patients with CMA. Indeed, in the publication by Franco et al. [9] this point was also considered with only a few samples from three studies reaching a level of β-lactoglobulin ≥ eliciting dose for 1% (ED01) of allergic individuals, and in no case did the samples contain beta-lactoglobulin in concentrations ≥ ED05 [12, 15, 16]. The probability therefore, according to this publication, of having enough β-lactoglobulin in breastmilk to trigger an allergic reaction has been estimated at 1 : 2,893 [9]. 

There are several problems with the aforementioned theoretical calculations. The first and most obvious is that the ED01 and ED05 have been based on IgE mediated allergy. Additionally, the testing method, maternal atopy, and the pathophysiology of non-IgE mediated allergy has not been considered in calculating potential reactions of cow’s milk protein through breastmilk. Studies have shown marked difference in human breastmilk between allergic versus non-allergic mothers, including the levels of short-chain fatty acids [17], which impact the gut microbiota of the infant. Additionally, protease inhibitors and apolipoproteins were present in much higher concentrations in breastmilk of allergic versus non-allergic mothers. These proteins have been suggested to be linked to both allergy and asthma [18]. Levels of β-lactoglobulin have also been found to be higher in the breastmilk of allergic mothers [19]. It has been hypothesized that maternal allergy status may impact on the digestion and absorption through a gut that may have increased permeability and therefore explain the higher level of β-lactoglobulin [9]. Differences in the composition of breastmilk in atopic versus non-atopic mothers have also been documented, which may also impact on how infants respond [12, 16, 20]. Finally, the authors of the recent scoping review have suggested that the quantitative evaluation of bovine β-lactoglobulin in human milk by ELISA could give rise to misleading interpretations. The inconsistency of the results obtained with immunochemical methods has been demonstrated in studies when samples previously positive for ELISA were not confirmed by high performance liquid chromatography and tandem mass spectrometry (HPLC-MS) [21]. Franco et al. [9] therefore suggest that a new analytical perspective for the detection of food allergens in human milk from intact proteins to digested peptide fragments is considered. 

All of the aforementioned studies discuss the presence of bovine β-lactoglobulin in breastmilk and the potential to cause non-IgE mediated CMA, but other allergens such as soya, wheat, and egg have been detected in breastmilk and therefore also have the potential to elicit non-IgE mediated food-allergic reactions, but data to substantiate this is sparce and primarily based on clinical experience [22, 23]. 

## Diagnosis 

The diagnosis of non-IgE mediated allergy in breastfed infants is further complicated, because many of the symptoms (i.e., diarrhea, constipation, regurgitation, colic-type pain) overlap with pediatric functional gastrointestinal disorders (FGID) and other commonly occurring childhood diseases. Vandenplas et al. [24] performed a worldwide assessment of FGID and found that 29% of infants suffered from regurgitation and 18% and 9% from constipation or diarrhea, respectively. The risk of overdiagnosis of non-IgE mediated CMA based on frequently used guidelines, which include these common symptoms, has been documented, with two recent publications [14, 25]. Neither of these publications however have acknowledged the fact that none of the guidelines suggest the application of symptoms without an allergy-focused history that includes a feeding history (including an assessment of the maternal diet) and considering this in context of the presenting symptoms, growth assessment of the infant, consideration of other diagnosis, and allergy testing (where appropriate). Whilst overdiagnosis is a risk, one also needs to acknowledge that underdiagnosis and delay in diagnosis still occurs as well [1, 26]. It is therefore critical, that the diagnostic process for a non-IgE mediated allergy starts with an allergy-focused history and eliminating differential diagnoses (Table 1). 

There are currently no recommended biomarkers for the diagnosis across the spectrum of non-IgE mediated allergies, which includes the breastfeeding infant presenting with these delayed symptoms. Some studies have proposed atopy patch testing to determine “delayed sensitisation” [29], however, this test has yielded conflicting results [30, 31, 32]. Additionally, inconsistencies with patch test procedure and solution use was highlighted by Gonzalez et al. [33] in a study in 2018 on non-IgE mediated CMA. As a result of the poor reproducibility of patch testing, international guidelines do not recommend patch testing as a routine test for the diagnosis of non-IgE mediated allergies [5, 34]. 

IgG and IgG4 testing have also been suggested to support a diagnosis of non-IgE mediated allergy, but these tests have little established clinical validity and their use is also not recommended in routine clinical practice [35], alongside tests of gastrointestinal permeability and mucosal inflammatory markers [36, 37]. In breastfed infants with FPIAP, fecal blood is a cardinal symptom; however, testing for fecal occult blood has also been shown to be non-specific and unreliable in both the diagnosis and resolution of symptoms in non-IgE mediated allergies [38]. 

Whilst the absence of specific IgE is an expected characteristic of non-IgE mediated food allergies, some children may present with overlapping disease and allergic co-morbidities associated with IgE sensitization (i.e., atopic dermatitis) [39, 40]. This has been reported in FPIES, eosinophilic esophagitis (EoE), and other recognized non-IgE mediated conditions where symptoms of atopic dermatitis, co-existing IgE mediated allergy and IgE mediated environmental allergies have been reported [40, 41]. An allergy-focused history should therefore guide the decision to perform targeted IgE/SPT [39, 40], but the interpretation of results requires careful consideration and may require oral food challenges (OFCs). 

Finally, endoscopy is commonly reported in research related to the diagnoses of various non-IgE mediated allergic conditions, including EoE, where this is an essential diagnostic tool. As this procedure can be technically difficult in young infants and requires full anesthesia, the EAACI position paper suggests that endoscopy only be performed when there is a strong suspicion of an alternative diagnosis or unremitting symptoms [27]. 

## Dietary management 

The maternal dietary elimination of offending allergens remains the mainstay for dietary management of non-IgE mediated allergies in breastfed infants. The most commonly reported allergen is cow’s milk, but a non-IgE mediated allergy in breastfed infants to soya, egg, and wheat has also been described [42, 43]. All of these allergens contribute significantly to the maternal diet and have the potential to negatively impact on the nutritional status of the breastfeeding mother [44] as well as the micronutrient content of breastmilk [45, 46]. It is therefore important to avoid unnecessary elimination diets and support the mother and child with nutrient optimization and targeted micronutrient supplements (i.e., vitamin D) where necessary. 

The length of a diagnostic elimination diet in non-IgE mediated food allergies varies according to guidelines but is usually between 2 and 4 weeks. Clinicians need to also take into account that circulating β-lactoglobulin can be detected up to 7 – 10 days after the elimination [9]. Lozinsky et al. [41] found that the majority of children with non-IgE mediated allergy had improvement of symptoms within 4 weeks; however, data were mainly based on non-breastfed children. All guidelines recommend the reintroduction or challenge for the confirmation and also assessment of tolerance [5, 34]. Outside of FPIES (where guidelines for challenges have been published) [47], almost no data exist on the reintroduction/challenge for confirmation of non-IgE mediated allergies. In the breastfed infant with non-IgE mediated allergies, an additional question is raised to whether reintroduction should occur through the mother’s diet or as a complementary food in the infant’s diet. There is no data to guide the decision for clinicians, and therefore the EAACI guidelines suggest that this decision should primarily be driven whether the child is still exclusively breastfed or whether complementary food/formula feed has been introduced [27]. In infants that have complementary foods/or some hypoallergenic formula as part of their diet, a direct reintroduction (through standard infant formula or foods) is likely to yield a less ambiguous result and lead to the faster expansion of tolerated foods, whereas the introduction through the maternal diet may take longer to provide an answer and only confirms a tolerance to low levels of β-lactoglobulin, but not cow’s milk and its derivates in normally consumed amounts. 

The updated iMAP guidelines on non-IgE mediated allergies, recommend in exclusively breastfed children that cow’s milk products should be reintroduced in the mother’s diet in previously consumed amounts over a 1-week period [48]. The latter recommendation was made based on data from Järvinen et al. [13] in a challenge-proven non-IgE mediated cow’s milk-allergic breastfed cohort. In this cohort, 16/17 infants reacted within a mean time of 21 hours (2 – 80 hours) after the reintroduction of cow’s milk in the lactating mother’s diet. Although it is well known that other allergens (i.e., egg, soya, wheat) do transfer through breast milk [22, 49], there are no studies on the reintroduction to confirm food allergies for these foods in breastfed infants, and therefore the protocol is heavily influenced by local practice. 

For breastfed infants with non-IgE mediated allergies who are already on solids, there is also very limited data. The iMAP guidelines provide a consensus-based milk ladder, which has been constructed on the existing data that heating and fermentation reduces the allergenicity of cow’s milk [50, 51, 52]. This step-wise reintroduction approach has gained popularity and, from what is known, is safe [53], but there is to date no data on efficacy and there is a need for standardization [54]. For other allergens (i.e., egg, soya, wheat) there is only one study by Meyer et al. [55] describing the re-introduction using a ladder approach. Whilst this study provides some guidance, it is heavily influenced by local practice. 

## Conclusion 

Non-IgE mediated allergies in breastfed infants remains a complex diagnosis that needs careful clinical consideration. There is the concern of both over- and underdiagnosis and most importantly the nutritional risk for the mother when unwarranted elimination diets are used. 

Table 2 summarizes the clinical practice points from this publication for clinicians working in this field for their day-to-day practice. 

## Funding 

None. 

## Conflict of interest 

Academic lectures for Danone/Nutricia, Mead Johnson, Nestle, and Abbott. Consultancy for Else Nutrition and on the CoMISS board for Nestle Health Science. 

**Table 1.** Non-IgE mediated allergic disorders, symptoms, and differential diagnoses (taken from the European Academcy for Allergy and Clinical Immunology position paper on the management of non-IgE mediated allergies in breastfed infants) [27].

Non-IgE mediated food allergy	Cardinal symptom	Additional symptoms	Differential diagnoses
Food protein-induced enterocolitis syndrome (FPIES) [28]	Acute FPIES: Vomiting 1 – 4 hours after ingestion Chronic FPIES: intermittent but progressive vomiting and diarrhea	Acute FPIES: pallor, lethargy, hypovolemia, hypotension, diarrhea Chronic FPIES: faltering growth	Gastro-esophageal reflux disease, sepsis, inborn errors of metabolism, pyloric stenosis, malrotation, intussusception, gastroenteritis with vomiting
Food protein-induced allergic proctocolitis (FPIAP)	Blood in stool	Occasional loose stools, mucous in the stools, painful flatus, anal excoriation	Gastrointestinal infections, fissures Infantile polyp, necrotizing enterocolitis, Meckel’s diverticulum, intussusception, infantile inflammatory bowel disease (rare)
Eosinophilic esophagitis (EoE)	Intermitted vomiting, abdominal discomfort, feeding difficulties	Faltering growth	Gastro-esophageal reflux of infancy, infantile inflammatory bowel disease
Food protein-induced constipation	Straining with soft stools	Fecal impaction, bloating, abdominal pain	Normal straining associated with infancy, idiopathic constipation, Hirschsprung’s disease
Food protein-induced gastro-esophageal reflux disease	Intermitted painful vomiting/regurgitation	Faltering growth, feeding difficulties, back-arching with pain	Gastro-esophageal reflux of infancy, acute gastroenteritis, food poisoning
Food protein-induced enteropathy (FPE)	Failure to thrive, diarrhea	Mucus and, bloating, abdominal pain, faltering growth, hypoalbunemia	Sepsis, congenital disaccharide malabsorption, metabolic disorders, chronic kidney disease, neglect, secondary lactose intolerance, chronic FPIES, autoimmune enteropathies, epithelial dysplasia syndromes, cystic fibrosis, immunodeficiencies and/or chronic infection, coeliac disease

**Table 2.** Clinical practice points on the diagnosis and management of non-IgE mediated allergy in breastfed infants.

An allergy-focused history is the cornerstone of the diagnosis of a non-IgE mediated allergy in breastfed infants.
Most research has been published on cow’s milk as common culprit food, but egg, soya, and wheat may also need to be considered.
Current guidelines suggest a trial of 2 – 4 weeks of maternal elimination diet (with symptom improvement) followed by the reintroduction of the offending allergen (with symptom deterioration) to confirm a diagnosis.
No biomarkers are recommended for non-IgE mediated allergies, unless there are symptoms of an IgE mediated allergy.
Unwarranted dietary elimination should be avoided, as this increases the nutritional risk for the mother and infant.
Targeted micronutrient supplementation should occur following a dietetic assessment.
There are no clear guidelines on how to re-introduce food allergens in breastfed infants, outside of data on the milk ladder and challenge guidelines for FPIES.

## References

[1] Schoemaker AA Sprikkelman AB Grimshaw KE Roberts G Grabenhenrich L Rosenfeld L Siegert S Dubakiene R Rudzeviciene O Reche M Fiandor A Papadopoulos NG Malamitsi-Puchner A Fiocchi A Dahdah L Sigurdardottir ST Clausen M Stańczyk-Przyłuska A Zeman K Mills EN Incidence and natural history of challenge-proven cow’s milk allergy in European children--EuroPrevall birth cohort. Allergy. 2015; 70: 963–972. 25864712 10.1111/all.12630

[2] Koletzko S Heine RG Grimshaw KE Beyer K Grabenhenrich L Keil T Sprikkelman AB Roberts G Non-IgE mediated cow’s milk allergy in EuroPrevall. Allergy. 2015; 70: 1679–1680. 26769086 10.1111/all.12681

[3] Caubet JC Szajewska H Shamir R Nowak-Węgrzyn A Non-IgE-mediated gastrointestinal food allergies in children. Pediatr Allergy Immunol. 2017; 28: 6–17. 27637372 10.1111/pai.12659

[4] Høst A Husby S Osterballe O A prospective study of cow’s milk allergy in exclusively breast-fed infants. Incidence, pathogenetic role of early inadvertent exposure to cow’s milk formula, and characterization of bovine milk protein in human milk. Acta Paediatr Scand. 1988; 77: 663–670. 3201972 10.1111/j.1651-2227.1988.tb10727.x

[5] Muraro A Werfel T Hoffmann-Sommergruber K Roberts G Beyer K Bindslev-Jensen C Cardona V Dubois A duToit G Eigenmann P Fernandez Rivas M Halken S Hickstein L Høst A Knol E Lack G Marchisotto MJ Niggemann B Nwaru BI Papadopoulos NG EAACI food allergy and anaphylaxis guidelines: diagnosis and management of food allergy. Allergy. 2014; 69: 1008–1025. 24909706 10.1111/all.12429

[6] Fiocchi A Brozek J Schünemann H Bahna SL von Berg A Beyer K Bozzola M Bradsher J Compalati E Ebisawa M Guzmán MA Li H Heine RG Keith P Lack G Landi M Martelli A Rancé F Sampson H Stein A World Allergy Organization (WAO) Diagnosis and Rationale for Action against Cow’s Milk Allergy (DRACMA) Guidelines. Pediatr Allergy Immunol. 2010; 21: 1–125. 10.1111/j.1399-3038.2010.01068.x 20618740

[7] Koletzko S Niggemann B Arato A Dias JA Heuschkel R Husby S Mearin ML Papadopoulou A Ruemmele FM Staiano A Schäppi MG Vandenplas Y Diagnostic approach and management of cow’s-milk protein allergy in infants and children: ESPGHAN GI Committee practical guidelines. J Pediatr Gastroenterol Nutr. 2012; 55: 221–229. 22569527 10.1097/MPG.0b013e31825c9482

[8] WHO. Regional Office for Europe Nutrition and Food Security, Geneva. 2001; 1-25.

[9] Franco C Fente C Sánchez C Lamas A Cepeda A Leis R Regal P Cow’s Milk Antigens Content in Human Milk: A Scoping Review. Foods. 2022; 11: 1183. 35741982 10.3390/foods11121783 PMC9222876

[10] Høst A Halken S Hypoallergenic formulas--when, to whom and how long: after more than 15 years we know the right indication! Allergy. 2004; 59: 45–52. 10.1111/j.1398-9995.2004.00574.x 15245358

[11] Jakobsson I Lothe L Ley D Borschel MW Effectiveness of casein hydrolysate feedings in infants with colic. Acta Paediatr. 2000; 89: 18–21. 10677051 10.1080/080352500750028997

[12] Axelsson I Jakobsson I Lindberg T Benediktsson B Bovine beta-lactoglobulin in the human milk. A longitudinal study during the whole lactation period. Acta Paediatr Scand. 1986; 75: 702–707. 3564937 10.1111/j.1651-2227.1986.tb10277.x

[13] Järvinen KM Mäkinen-Kiljunen S Suomalainen H Cow’s milk challenge through human milk evokes immune responses in infants with cow’s milk allergy. J Pediatr. 1999; 135: 506–512. 10518086 10.1016/s0022-3476(99)70175-7

[14] Munblit D Perkin MR Palmer DJ Allen KJ Boyle RJ Assessment of Evidence About Common Infant Symptoms and Cow’s Milk Allergy. JAMA Pediatr. 2020; 174: 599–608. 32282040 10.1001/jamapediatrics.2020.0153

[15] Monti JC Mermoud AF Jollès P Anti-bovine beta-lactoglobulin antibodies react with a human lactoferrin fragment and bovine beta-lactoglobulin present in human milk. Experientia. 1989; 45: 178–180. 2920804 10.1007/BF01954867

[16] Høst A Husby S Hansen LG Osterballe O Bovine beta-lactoglobulin in human milk from atopic and non-atopic mothers. Relationship to maternal intake of homogenized and unhomogenized milk. Clin Exp Allergy. 1990; 20: 383–387. 2376021 10.1111/j.1365-2222.1990.tb02798.x

[17] Stinson LF Gay MCL Koleva PT Eggesbø M Johnson CC Wegienka G du Toit E Shimojo N Munblit D Campbell DE Prescott SL Geddes DT Kozyrskyj AL Human Milk From Atopic Mothers Has Lower Levels of Short Chain Fatty Acids. Front Immunol. 2020; 11: 1427. 32903327 10.3389/fimmu.2020.01427 PMC7396598

[18] Hettinga KA Reina FM Boeren S Zhang L Koppelman GH Postma DS Vervoort JJ Wijga AH Difference in the breast milk proteome between allergic and non-allergic mothers. PLoS One. 2015; 10: e0122234. 25798592 10.1371/journal.pone.0122234 PMC4370490

[19] Dekker PM Boeren S Wijga AH Koppelman GH Vervoort JJM Hettinga KA Maternal Allergy and the Presence of Nonhuman Proteinaceous Molecules in Human Milk. Nutrients. 2020; 12: E1169. 32331315 10.3390/nu12041169 PMC7230597

[20] Matangkasombut P Padungpak S Thaloengsok S Kamchaisatian W Sasisakulporn C Jotikasthira W Benjaponpitak S Manuyakorn W Detection of β-lactoglobulin in human breast-milk 7 days after cow milk ingestion. Paediatr Int Child Health. 2017; 37: 199–203. 28222656 10.1080/20469047.2017.1289310

[21] Pastor-Vargas C Maroto AS Díaz-Perales A Villaba M Casillas Diaz N Vivanco F Cuesta-Herranz J Sensitive detection of major food allergens in breast milk: first gateway for allergenic contact during breastfeeding. Allergy. 2015; 70: 1024–1027. 25952012 10.1111/all.12646

[22] Franke AA Halm BM Custer LJ Tatsumura Y Hebshi S Isoflavones in breastfed infants after mothers consume soy. Am J Clin Nutr. 2006; 84: 406–413. 16895891 10.1093/ajcn/84.1.406

[23] Cant A Marsden RA Kilshaw PJ Egg and cows’ milk hypersensitivity in exclusively breast fed infants with eczema, and detection of egg protein in breast milk. Br Med J (Clin Res Ed). 1985; 291: 932–935. 10.1136/bmj.291.6500.932 PMC1417254 3929969

[24] Vandenplas Y Abkari A Bellaiche M Benninga M Chouraqui JP Çokura F Harb T Hegar B Lifschitz C Ludwig T Miqdady M de Morais MB Osatakul S Salvatore S Shamir R Staiano A Szajewska H Thapar N Prevalence and Health Outcomes of Functional Gastrointestinal Symptoms in Infants From Birth to 12 Months of Age. J Pediatr Gastroenterol Nutr. 2015; 61: 531–537. 26308317 10.1097/MPG.0000000000000949 PMC4631121

[25] Vincent R MacNeill SJ Marrs T Craven J Logan K Flohr C Lack G Radulovic S Perkin MR Ridd MJ Frequency of guideline-defined cow’s milk allergy symptoms in infants: Secondary analysis of EAT trial data. Clin Exp Allergy. 2022; 52: 82–93. 34877731 10.1111/cea.14060

[26] Sladkevicius E Nagy E Lack G Guest JF Resource implications and budget impact of managing cow milk allergy in the UK. J Med Econ. 2010; 13: 119–128. 20092426 10.3111/13696990903543242

[27] Meyer R Chebar Lozinsky A Fleischer DM Vieira MC Du Toit G Vandenplas Y Dupont C Knibb R Uysal P Cavkaytar O Nowak-Wegrzyn A Shah N Venter C Diagnosis and management of Non-IgE gastrointestinal allergies in breastfed infants-An EAACI Position Paper. Allergy. 2020; 75: 14–32. 31199517 10.1111/all.13947

[28] Mehr S Frith K Barnes EH Campbell DE Food protein-induced enterocolitis syndrome in Australia: A population-based study, 2012-2014. J Allergy Clin Immunol. 2017; 140: 1323–1330. 28427879 10.1016/j.jaci.2017.03.027

[29] Cudowska B Kaczmarski M Atopy patch test in the diagnosis of food allergy in children with gastrointestinal symptoms. Adv Med Sci. 2010; 55: 153–160. 21084256 10.2478/v10039-010-0038-z

[30] Niggemann B Reibel S Roehr CC Felger D Ziegert M Sommerfeld C Wahn U Predictors of positive food challenge outcome in non-IgE-mediated reactions to food in children with atopic dermatitis. J Allergy Clin Immunol. 2001; 108: 1053–1058. 11742288 10.1067/mai.2001.120192

[31] Järvinen KM Caubet JC Sickles L Ford LS Sampson HA Nowak-Węgrzyn A Poor utility of atopy patch test in predicting tolerance development in food protein-induced enterocolitis syndrome. Ann Allergy Asthma Immunol. 2012; 109: 221–222. 22920080 10.1016/j.anai.2012.06.020 PMC3586209

[32] Lucarelli S Di Nardo G Lastrucci G D’Alfonso Y Marcheggiano A Federici T Frediani S Frediani T Cucchiara S Allergic proctocolitis refractory to maternal hypoallergenic diet in exclusively breast-fed infants: a clinical observation. BMC Gastroenterol. 2011; 11: 82. 21762530 10.1186/1471-230X-11-82 PMC3224143

[33] Gonzaga TA Alves FA Cheik MFA de Barros CP Rezende ERMA Segundo GRS Low efficacy of atopy patch test in predicting tolerance development in non-IgE-mediated cow’s milk allergy. Allergol Immunopathol (Madr). 2018; 46: 241–246. 29031891 10.1016/j.aller.2017.07.001

[34] Boyce JA Assa’ad A Burks AW Jones SM Sampson HA Wood RA Plaut M Cooper SF Fenton MJ Arshad SH Bahna SL Beck LA Byrd-Bredbenner C Camargo CA Eichenfield L Furuta GT Hanifin JM Jones C Kraft M Levy BD Guidelines for the diagnosis and management of food allergy in the United States: report of the NIAID-sponsored expert panel. J Allergy Clin Immunol. 2010; 126: S1–S58. 21134576 10.1016/j.jaci.2010.10.007 PMC4241964

[35] Bock SA AAAAI support of the EAACI Position Paper on IgG4. J Allergy Clin Immunol. 2010; 125: 1410. 10.1016/j.jaci.2010.03.013 20451986

[36] Campeotto F Butel MJ Kalach N Derrieux S Aubert-Jacquin C Barbot L Francoual C Dupont C Kapel N High faecal calprotectin concentrations in newborn infants. Arch Dis Child Fetal Neonatal Ed. 2004; 89: F353–F355. 15210674 10.1136/adc.2002.022368 PMC1721713

[37] Poullis A Foster R Northfield TC Mendall MA Review article: faecal markers in the assessment of activity in inflammatory bowel disease. Aliment Pharmacol Ther. 2002; 16: 675–681. 11929384 10.1046/j.1365-2036.2002.01196.x

[38] Feuille E Nowak-Węgrzyn A Definition, etiology, and diagnosis of food protein-induced enterocolitis syndrome. Curr Opin Allergy Clin Immunol. 2014; 14: 222–228. 24686276 10.1097/ACI.0000000000000055 PMC4011631

[39] Bardana EJ Immunoglobulin E Immunoglobulin E- (IgE) and non-IgE-mediated reactions in the pathogenesis of atopic eczema/dermatitis syndrome (AEDS). Allergy. 2004; 59: 25–29. 15245353 10.1111/j.1398-9995.2004.00565.x

[40] Meyer R Fleming C Dominguez-Ortega G Lindley K Michaelis L Thapar N Elawad M Chakravarti V Fox AT Shah N Manifestations of food protein induced gastrointestinal allergies presenting to a single tertiary paediatric gastroenterology unit. World Allergy Organ J. 2013; 6: 13. 23919257 10.1186/1939-4551-6-13 PMC3828665

[41] Chebar Lozinsky A Time to symptom improvement using elimination diets in non-IgE mediated gastrointestinal food allergies. Pediatr Allergy Immunol. 2015; 2: 317–329. 10.1111/pai.12404 25963794

[42] Hill DJ Roy N Heine RG Hosking CS Francis DE Brown J Speirs B Sadowsky J Carlin JB Effect of a low-allergen maternal diet on colic among breastfed infants: a randomized, controlled trial. Pediatrics. 2005; 116: e709–e715. 16263986 10.1542/peds.2005-0147

[43] Atanaskovic-Markovic M Refractory proctocolitis in the exclusively breast-fed infants. Endocr Metab Immune Disord Drug Targets. 2014; 14: 63–66. 24450450 10.2174/1871530314666140121145800

[44] Kramer MS Kakuma R Maternal dietary antigen avoidance during pregnancy or lactation, or both, for preventing or treating atopic disease in the child. Cochrane Database Syst Rev. 2006; 3:CD000133. 10.1002/14651858.CD000133.pub2 16855951

[45] Bravi F Wiens F Decarli A Dal Pont A Agostoni C Ferraroni M Impact of maternal nutrition on breast-milk composition: a systematic review. Am J Clin Nutr. 2016; 104: 646–662. 27534637 10.3945/ajcn.115.120881

[46] Thomassen RA Kvammen JA Eskerud MB Júlíusson PB Henriksen C Rugtveit J Iodine Status and Growth In 0-2-Year-Old Infants With Cow’s Milk Protein Allergy. J Pediatr Gastroenterol Nutr. 2017; 64: 806–811. 27741063 10.1097/MPG.0000000000001434

[47] Nowak-Węgrzyn A Chehade M Groetch ME Spergel JM Wood RA Allen K Atkins D Bahna S Barad AV Berin C Brown Whitehorn T Burks AW Caubet J-C Cianferoni A Conte M Davis C Fiocchi A Grimshaw K Gupta R Hofmeister B International Consensus Guidelines for the Diagnosis and Management of Food Protein-Induced Enterocolitis Syndrome: Workgroup Report of the Adverse Reactions to Foods Committee, American Academy of Allergy, Asthma, and Immunology. J Allergy Clin Immunol. 2017; 139: 1111–1126. 28167094 10.1016/j.jaci.2016.12.966

[48] Venter C Brown T Meyer R Walsh J Shah N Nowak-Węgrzyn A Chen TX Fleischer DM Heine RG Levin M Vieira MC Fox AT Better recognition, diagnosis and management of non-IgE-mediated cow’s milk allergy in infancy: iMAP-an international interpretation of the MAP (Milk Allergy in Primary Care) guideline. Clin Transl Allergy. 2017; 7:26. 28852472 10.1186/s13601-017-0162-y PMC5567723

[49] Palmer DJ Gold MS Makrides M Effect of cooked and raw egg consumption on ovalbumin content of human milk: a randomized, double-blind, cross-over trial. Clin Exp Allergy. 2005; 35: 173–178. 15725188 10.1111/j.1365-2222.2005.02170.x

[50] Nowak-Wegrzyn A Tolerance to extensively heated milk in children with cow’s milk allergy. J Allergy Clin Immunol. 2008; 122: 342–347. 18620743 10.1016/j.jaci.2008.05.043

[51] Alessandri C Sforza S Palazzo P Lambertini F Paolella S Zennaro D Rafaiani C Ferrara R Bernardi ML Santoro M Zuzzi S Giangrieco I Dossena A Mari A Tolerability of a fully maturated cheese in cow’s milk allergic children: biochemical, immunochemical, and clinical aspects. PLoS One. 2012; 7: e40945. 22829901 10.1371/journal.pone.0040945 PMC3400663

[52] Uncuoglu A Yologlu N Simsek IE Uyan ZS Aydogan M Tolerance to baked and fermented cow’s milk in children with IgE-mediated and non-IgE-mediated cow’s milk allergy in patients under two years of age. Allergol Immunopathol (Madr). 2017; 45: 560–566. 28720381 10.1016/j.aller.2017.02.008

[53] Athanasopoulou P Deligianni E Dean T Dewey A Venter C Use of baked milk challenges and milk ladders in clinical practice: a worldwide survey of healthcare professionals. Clin Exp Allergy. 2017; 47: 430–434. 28109173 10.1111/cea.12890

[54] Venter C Meyer R Ebisawa M Athanasopoulou P Mack DP Food allergen ladders: A need for standardization. Pediatr Allergy Immunol. 2022; 33: e13714. 34882843 10.1111/pai.13714

[55] Meyer R The Challenge of Home Allergen Re-introductions Using the Ladder Approach in Children With Non-IgE Mediated Gastrointestinal Food Allergy. Front Allergy. 2021; 721686. 10.3389/falgy.2021.721686 PMC8974734 35386976

